# From F. W. Dolbeare

**Published:** 1884-04

**Authors:** F. W. Dolbeare

**Affiliations:** 101 South Oxford St., Brooklyn, N. Y.


101 South Oxford St.,
Brooklyn, N. Y., March 10, 1884.

*Editor Independent Practitioner:*

Recognizing the rapidity and accuracy with which the dental engine replaces all similar operations by hand, I have always been an outspoken advocate in its behalf. Since the introduction of the Elliott suspension engine, I have had one in continuous operation, and until recently it has given perfect satisfaction. Modern and improved hand-pieces, together with the worn-out condition of my engine, led me to investigate and to consider the best way to supply myself with good and efficient means.

Having used the suspension action so long, I was unwilling to entirely part with it, and yet I was not ignorant of its faults, nor so prejudiced in its favor that I could not discern the merits of other and different manufactures.

The position of my operating chair is against a party-wall, on the left hand side; the ceiling is twelve feet high, thus being favorable for my modification, and disadvantageous for the suspension. Against the party-wall, on a line with the chair's head-rest, and six feet from the floor, I fastened a wall plate, and upon that attached a piece of bronze gas-pipe twenty inches long and three-quarters of an inch in diameter, bringing the outer end directly over my chair. To this is attached a second pipe, of smaller diameter and same length, by a swivel joint. At this juncture a couple of pulley wheels are attached, the small brass rod on which they run being screwed into the joint. At the outer extremity of the second piece of pipe, running into the bore and fastened in by a set-screw, is the cable part of the S. S. White Improved Dental Engine. For the motor power I use the Johnston suspension. The driving cord runs over the two pulley wheels, along the second piece of pipe to the end, and over the set-wheel of the flexible cable.

In this modification I have the running power and firmness of the standard engine, and the "out-of-the-wayness" of the suspension.

Thinking that others may possibly find the suggestion of some value, I send it to you for their benefit.

Very truly yours,

F. W. Dolbeare.

